# P-1893. Evaluation of Infectious Disease Consultation on Outpatient Antimicrobial Therapy (OPAT) Referral Volume and Outcomes

**DOI:** 10.1093/ofid/ofae631.2054

**Published:** 2025-01-29

**Authors:** Nicole Slain, Ryan P Mynatt, Kathryn Ruf, Evelyn Villacorta Cari, Armaghan-E Rehman Mansoor, Donna R Burgess, Alisha Clemons, Ashley Logan

**Affiliations:** University of Kentucky HealthCare, Lexington, Kentucky; University of Kentucky, Lexington, KY; University of Kentucky HealthCare, Lexington, Kentucky; University of Kentucky, Lexington, KY; University of Kentucky, Lexington, KY; UK HealthCare, Lexington, KY; University of Kentucky, Lexington, KY; University of Kentucky HealthCare, Lexington, Kentucky

## Abstract

**Background:**

Institutional experience with OPAT is increasingly reported. However, little is known about the referral process across the continuum of care and its impact on OPAT volume and patient outcomes. The purpose of this study is to characterize OPAT referral trends from a multi-service line infectious diseases (ID) group and its impact on end of therapy patient outcomes.

**Figure:**
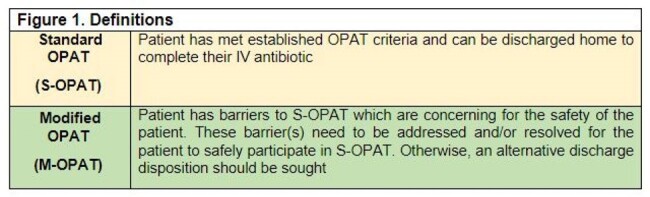

**Methods:**

This retrospective analysis included adult patients referred to the OPAT program from either of the two system hospitals and seen by any of the five inpatient consult teams or the ambulatory ID clinic between June 2021 and July 2022. Patients were identified via a data extraction from the electronic health record by the institution’s Healthcare Analytics Team or Center for Clinical and Translational Sciences. OPAT patients were identified via the OPAT Electronic Data Capture (REDCap) database.

**Figure:**
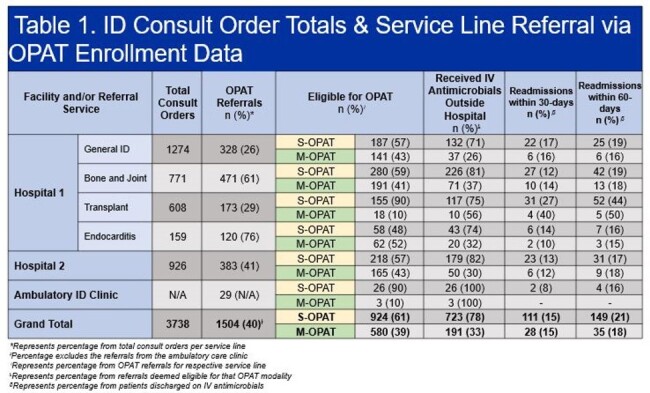

**Results:**

A total of 3738 ID consult orders were placed on 2708 patients, dispersed between four consult teams at hospital 1 (Table 1). The OPAT program received 1504 referrals, 1475 (98.1%) inpatient referrals and 29 (1.9%) outpatient. A total of 914 patients were discharged to receive IV antimicrobials, 723 (78.2%) and 191 (32.9%) (p < 0.00001), for standard OPAT (S-OPAT) and modified OPAT (M-OPAT), respectively (Figure 1). End of therapy outcomes were recorded on 798 (87.3%) patients. The Transplant service had the highest percentage of referrals eligible for S-OPAT (90%); however, it also saw the highest 60-day readmission rate (36.2%) (Table 1). There was no statistically significant difference (p = 0.281) in proportion of referrals with end of treatment failure within the ID services at hospital 1. However, therapy extension was more frequent in the Bone and Joint and General ID teams (p < 0.001). Hospital 2 had more patients that were unable to complete their antimicrobial therapy (14.6%) (Table 3).

**Figure:**
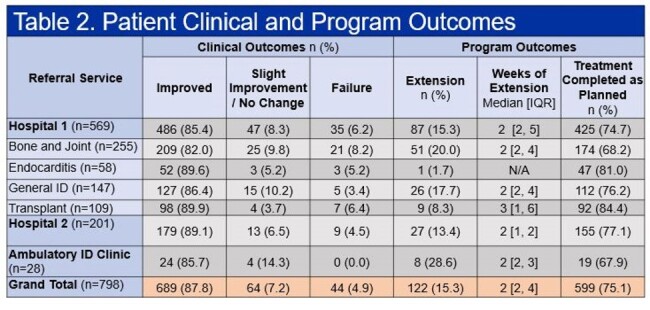

**Conclusion:**

Our analysis of patients seen by ID consult services demonstrated a significant portion were ultimately referred for OPAT. Differences in referral rates were noted between service lines, along with differences in 30 and 60-day readmission rates. Overall, end of therapy and clinical outcomes were fairly similar throughout the groups.

**Figure:**
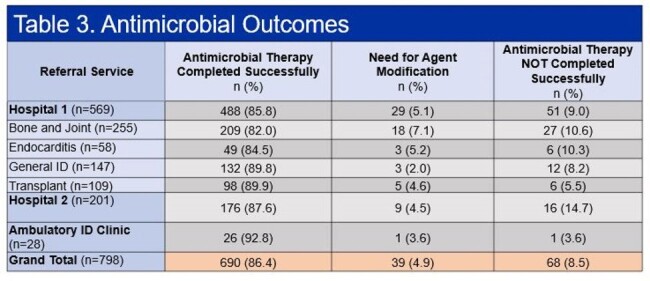

**Disclosures:**

Alisha Clemons, APRN, Gilead Sciences: Honoraria

